# Molecular diagnosis of *Theileria *and *Babesia *species infecting cattle in Northern Spain using reverse line blot macroarrays

**DOI:** 10.1186/1746-6148-2-16

**Published:** 2006-05-09

**Authors:** Josune García-Sanmartín, Daniel Nagore, Ana L García-Pérez, Ramón A Juste, Ana Hurtado

**Affiliations:** 1 Department of Animal Health, NEIKER – Instituto Vasco de Investigación y Desarrollo Agrario, Berreaga 1, 48160 Derio, Bizkaia, Spain; 2 Proteomika, S.L., Parque Tecnologico de Zamudio, Edif. 801, 48160 Derio, Bizkaia, Spain

## Abstract

**Background:**

Piroplasmosis in cattle is caused by tick-borne haemoprotozoan parasites of the genera *Theileria *and *Babesia*. Molecular detection techniques offer higher sensitivity and specificity than microscopy examination methods and serological tests. A reverse line blot (RLB) macroarray that included generic and species-specific probes for *Theileria annulata*, *Theileria buffeli*, *Babesia bovis*, *Babesia bigemina*, *Babesia divergens *and *Babesia major *was used to study the presence and identity of the piroplasm species infecting 263 bovine blood samples from 79 farms, most of them in Northern Spain. Microscopy examination of blood smears and haematology were also performed whenever possible to identify animals with parasitaemia.

**Results:**

RLB hybridisation identified infection in 54.0% of the samples, whereas only 28.8% were positive by microscopy examination. The most frequently found species was *T. buffeli*, present in 42.6% of the samples. *T. annulata *was found in 22 samples (8.4%) from 12 farms, including 9 farms (14 samples) located in Northern Spain where presence of the vector is not very common. *Babesia *infections were less frequently detected: *B. major *was found in 3.0% of the samples, *B. bigemina *in 2.7%, *B. bovis *in 2.3% and *B. divergens *in 1.1%. Mixed infections were detected in 14 samples, accounting for six different combinations of species.

**Conclusion:**

This is the first report in which *B. major *and *B. divergens *have been detected in Spain using molecular identification techniques and the first time that *B. bovis *has been detected in Northern Spain. The detection of *T. annulata *in Northern Spain suggests that the distribution of Mediterranean theileriosis might be changing. Samples with positive RLB hybridisation but negative microscopy had haematology values within the normal ranges suggesting that they corresponded to chronic carriers that may serve as reservoirs of the infection. In this sense, sensitive and specific laboratorial tests like RLB that clearly identify the parasite and can detect subclinical infections are essential to establish good control measures.

## Background

Piroplasmosis in cattle is caused by tick-borne haemoprotozoan parasites comprising several *Theileria *and *Babesia *species [1,2]. These diseases are of serious health and economic concern in several parts of the world, including tropical and temperate countries. In Spain the main malignant piroplasm species causing severe theileriosis signs is *Theileria annulata*, with infections described in Southern and Mediterranean areas [3-6], where the tick vector (*Hyalomma *sp.) is present [7,8]. However, the most widespread theileria is *Theileria buffeli*, which is in general considered a benign species. Regarding *Babesia *spp., several pathogenic species have been reported in Central and Southern Spain (*B. major*, *B. bovis*, *B. divergens *and *B. bigemina*) [7], though only *B. bigemina *and *B. bovis *have been detected in these areas in recent epidemiological studies using molecular methods [3,9]. Different weather conditions in different parts of Spain determine tick vector distribution, and therefore, the incidence of piroplasmosis. Unlike the dry and hot Mediterranean climate of eastern and southern parts, Northern Spain covers two different climatic zones: an Atlantic climate in the northern coast with frequent rainfall and mild temperatures, and a Continental Mediterranean climate in the interior central plateau with colder winters and warmer summers. Hence, the main tick species infesting cattle in Northern Spain are *Ixodes ricinus*,* Haemaphysalis punctata*, *Dermacentor *spp. and *Rhipicephalus bursa *[10,11]. Moving in a southerly direction, ticks of the genus *Hyalomma *become more prevalent, and similarly, other tick species such as *I. ricinus *and *Dermacentor reticulatus *become nearly absent [7,8]. Therefore, a different situation regarding bovine piroplasms diversity would be expected in this northern area compared to other parts of Spain. However, there are no studies on bovine piroplasmosis in Northern Spain using molecular identification techniques.

Laboratorial diagnosis of clinical infection by piroplasms in cattle is usually based on the detection of the parasite in Giemsa-stained blood smears. Carrier animals, in which low numbers of erythrocytes remain infected, are important contributors to the transmission of the infection by tick bites. Hence, detection of piroplasms in carrier animals is very important to control the infection. However, detection of piroplasms by microscopy is not easy and it is generally not possible to discriminate pathogenic from non-pathogenic species that may occur simultaneously within the same host. Although serological tests can be used to detect circulating antibodies, cross-reactivity with antibodies directed against other species of piroplasms has been reported [12]. Moreover, antibodies tend to disappear in long-term carriers, whereas piroplasms persist. Therefore, animals with a negative serological test can still be the source of the infection and infect ticks. Several PCR-based diagnosis procedures for the identification of these parasites have been developed [13-16]. However, increased sensitivity and specificity can be achieved by combining PCR with a specific hybridisation by means of reverse line blot (RLB), a macroarray that is also capable of identifying mixed infections [9,17-20].

The aim of this study was to investigate the presence and identity of piroplasm species infecting the bovine blood samples with suspicion of piroplasmosis submitted to the Diagnostic Service of our Institute from Northern Spain in recent years using reverse line blot (RLB) macroarrays.

## Results

Microscopy examination of blood samples identified intracellular forms morphologically compatible with piroplasms in 28.8% of them (Table 1), 82.5% corresponding to *Theileria *spp. and 17.5% to *Babesia *spp. Piroplasms were detected by microscopy in 37.8 % of the animals with clinical signs compatible with piroplasmosis and in 24.6% of those with unspecific signs. In samples with *Babesia *infection red cell parameters (erythrocyte count 4.18 ± 1.19 × 10^6 ^mm^-3^, PCV 0.19 ± 0.039 l/l, and haemoglobin 5.9 ± 1.54 g/dl) were significantly lower (P < 0.05) than in those with *Theileria *spp. (erythrocyte count 5.58 ± 0.50 × 10^6 ^mm^-3^, PCV 0.27 ± 0.023 l/l, and haemoglobin 9.7 ± 0.79 g/dl) or negative (erythrocyte count 5.85 ± 1.41 ×10^6 ^mm^-3^, PCV 0.30 ± 0.096 l/l, and haemoglobin 10.4 ± 1.78 g/dl). Red cell parameters in samples with *Theileria *spp. were lower than in negative samples but differences were not significant. Samples with mixed infections (*Babesia *spp. and *Theileria *spp.) presented intermediate values (erythrocyte count 5.01 ± 1.53 × 10^6 ^mm^-3^, PCV 0.25 ± 0.065 l/l, and haemoglobin 8.4 ± 2.11 g/dl) that were significantly lower than in negative samples in terms of PCV. Leukocyte countings were also significantly lower (4.46 ± 0.96 × 10^3 ^mm^-3^) in animals with *Babesia *than in negative (7.18 ± 0.56 × 10^3 ^mm^-3^) or *Theileria*-infected animals (7.38 ± 0.77 × 10^3 ^mm^-3^) (P < 0.05).

**Figure 1:**
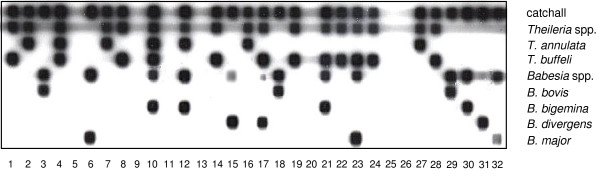
**Reverse line blot macroarray of the bovine blood samples and control clones**. Oligonucleotide probes are indicated in rows, and samples are applied in columns as follows: lanes 1–24, field samples; lane 25, genomic DNA of uninfected cow; lane 26, negative control; lane 27, *T. annulata *18S rRNA gene clone; lane 28, *T. buffeli *18S rRNA gene clone; lane 29, *B. bovis *18S rRNA gene clone; lane 30, *B. bigemina *18S rRNA gene clone; lane 31, *B. divergens *18S rRNA gene clone; lane 32, *B. major *18S rRNA gene clone.

142 of the blood samples analysed hybridised with at least one of the species-specific probes of the RLB array, finding positive signals with all six probes (Fig. 1). Thus, positive samples increased to 54.0% when analysed by RLB (Table 1), with piroplasms being detected in 60.9% of the animals with suspicion of piroplasmosis and in 50.0% of those with unspecific signs. *T. buffeli *was the most frequently found species being present in 42.6% of the samples (112/263), mainly as a single infection (90.2%, 101/112) but also in mixed infections (9.8%, 11/112). The highly pathogenic species *T. annulata *was second in detection with 8.4% of the samples positive (22/263), in 15 of them as a single infection (68.2%) and as part of mixed infections in the remaining 7 samples (31.8%). *T. annulata *was found in 12 farms distributed throughout Spain, including the 3 farms from the endemic regions (2 from Southern Spain and 1 from the Mediterranean area) that accounted for 8 animals with single infection. The remaining 9 farms positive to *T. annulata *were in Northern Spain and they appeared both as single and mixed infections. In six of these farms only 1 or 2 animals were analysed, being all of them positive to *T. annulata *(as a mixed infection in half of them). In the other three farms the number of animals sampled varied among 4 (1 animal positive to *T. annulata*), 5 (2 positive) and 13 (3 positive), and other piroplasm species besides *T. annulata *were also found in all three farms.

Presence of *Babesia *species was less frequently detected, with 24 samples positive (9.1%) either as single or mixed infection (Table 1). The species most frequently found was *B. major*, present in 8 animals (3.0%) from 3 farms. In five samples it was found as a single infection whereas in the remaining 3 it appeared combined with *T. buffeli*. *B. bigemina *was detected in 7 animals (2.7%) from 5 farms and it always appeared as part of a mixed infection with one or both species of *Theileria*. Conversely, *B. bovis *was only found as a single infection present in 6 animals (2.2%) from 3 farms from Northern Spain. Finally, *B. divergens *was only found in 3 samples (1.1%) from 3 farms, in one animal it occurred as a single infection while in the other two, *B. divergens *was detected along with *T. buffeli*.

There was no significant difference in the percentage of animals infected with *T. annulata *or *T. buffelli *between the group of animals with clinical suspicion of piroplasmosis and those with unspecific signs. However, detection of *B. major *and *B. bovis *was significantly associated to the group of animals with clinical suspicion of piroplasmosis (P < 0.05).

Mixed infections were detected in a total of 14 samples, accounting for six different combinations of species (Table 1). One of them included three species and the remaining five were composed of a combination of two species. Whereas *T. buffeli *and *T. annulata *were found together in 4 samples (2 in combination with *B. bigemina*), no mixed infections with two *Babesia *species were detected. *T. buffeli*, the most prevalent species, was found in combination with all the other species except with *B. bovis*, which was only found in single infections.

When compared with microscopy, RLB detected a positive signal in 40.0% of the samples with negative microscopy (72 samples), identifying *T. buffeli *in 75.0% of them,*T. annulata *in 15.3%, and several species of *Babesia *in 13.9%. However, 10 samples positive by microscopy were not detected by RLB analysis. When looking only to the RLB results of the animals with negative microscopy (subclinical infections), the lowest red cell parameters were found in one animal infected with *B. major *(n = 1, erythrocyte count 3.02 × 10^6 ^mm^-3^, PCV 0.17 l/l, and haemoglobin 5.23 g/dl) and the animals infected with *B. bovis *(n = 6, erythrocyte count 5.12 ± 1.04 × 10^6 ^mm^-3^, PCV 0.24 ± 0.042 l/l, and haemoglobin 7.02 ± 1.95 g/dl), whereas animals infected with species of *Theileria *had lower values than negative animals but within the normal ranges.

Although the number of samples submitted from each farm was in general quite small (more than one sample was only submitted from 52% of the farms), 2 pathogenic species (*i.e. *different to *T. buffeli*) were found in 5 farms, located all of them in Northern Spain. *T. annulata *was present in all five, along with *B. bigemina *in four of them and with *B. bovis *in the fifth.

## Discussion

The technique used in this study allowed the simultaneous detection and identification of different bovine *Theileria *and *Babesia *species using oligonucleotide probes whose specificity has been previously determined [9,17,19]. Moreover, the combination of a generic *Babesia *and *Theileria *PCR targeting the V4 region of the 18S rRNA gene and a hybridisation with specific probes provided high sensitivity [9,19]. Since detection of the parasite in Giemsa-stained blood smears is the technique that has been traditionally used for diagnosis of piroplasmosis, whenever possible RLB and microscopy examination were performed in parallel. Besides, positive microscopy allowed us to identify animals with parasitaemia. Thus, RLB hybridisation detected piroplasms in 72 samples that were negative by microscopy examination reaching a total of 54.0% of positive samples, while only 28.8% could be detected by microscopy. In any case, and even though RLB was significantly more sensitive than microscopy, piroplasms were detected by microscopy in 10 samples negative by RLB. This discrepancy could be due to subjective interpretation of microscopy examination or bad conditions of the samples (e.g. haemolysis or presence of clots). Whatever the reason, the inclusion of the catchall and genus-specific probes guarantees that this discrepancy cannot be ascribed to infection with new piroplasm species.

*T. buffeli *was the most prevalent species with 42.6% of the samples positive by RLB. This value is much lower than that reported in Eastern Spain using the same technology [3], but higher than in Northeastern Portugal [21]. *T. buffeli *was mainly found as a single infection, but it was also the most common species found in mixed infections. Although Stockham and cols. [22] described a case of clinical disease associated to *T. buffeli *infection, this species is generally considered as non-pathogenic. The results obtained in this study would confirm this idea, since samples with organisms morphologically compatible with *Theileria *spp. by microscopy examination and identified as *T. buffeli *by RLB presented red cell parameters within the normal ranges. However, this does not exclude the possibility that subclinical infections with *T. buffeli *might cause a production decrease. Further research is therefore needed to establish the significance and effect on bovine production of this highly prevalent species. Conversely, *T. annulata *is clearly recognised as a very pathogenic theileria that has been mainly described in temperate areas as the causative agent of Mediterranean theileriosis. In Spain most of the cases are generally restricted to southern and eastern parts of the country, where the tick vector (*Hyalomma *spp.) is endemic [4,7,8]. In this study *T. annulata *was found in 22 samples from 12 farms, 3 in the endemic regions and 9 in Northern Spain. The lower incidence of *T. annulata *compared to *T. buffeli *(8.4% *vs. *42.6%) is in agreement with the different geographic distribution of the tick vectors associated to each species. Thus, whereas the vector of *T. buffeli *(*Haemaphysalis *spp.) is quite common in Northern Spain [23,24], ticks of the genus *Hyalomma *seem to be endemic of southern and eastern parts of the country and reports on its presence in Northern Spain are only sporadic [7,8,25]. In this sense, recent data (S. Jiménez, personal communication) have revealed the recent emergence of *Hyalomma *ticks in cattle in La Rioja (a province in Northern Spain), suggesting that the distribution of Mediterranean theileriosis might be changing, probably due to changes in climatic conditions and vegetation that affect vector distribution.

Infection with *Babesia *species accounted for 9.1% of the samples corresponding to 3.0% of *B. major*, 2.7% of *B. bigemina*, 2.2% of *B. bovis *and 1.1% of *B. divergens*. However, this is the first time that *B. major *and *B. divergens *are detected in Spain using molecular techniques. In previous reports species identification had been based on morphological examinations by microscopy. Tick vectors associated to these species seem to be *H. punctata *and *I. ricinus*, respectively [26], which are very abundant in Northern Spain [23,24]. *B. bovis *is regarded as the most pathogenic bovine *Babesia *species and so far in Spain it had only been reported in Balearic Islands (Eastern Spain) [27], although it had been identified before in Southern and Western Spain by microscopy examination [7]. *B. bovis *is transmitted by ticks of three genera (*Ixodes*, *Rhipicephalus *and *Boophilus*) [26], although only the first two are found in Northern Spain. In our study we found 6 positive samples from 3 farms in Northern Spain with a history of piroplasmosis. In two farms *B. bovis *was detected in 3 of 4 samples, and 2 out of 6, respectively. In the third farm, samples had been submitted to the laboratory at four different time points (four samples) throughout 3 years with reports of unspecific clinical signs. In addition to *B. bovis*, *T. annulata *and *T. buffeli *were also detected in one animal each, suggesting that different piroplasms were circulating within that farm. However, in none of the 6 positive samples was *B. bovis *found in a mixed infection.

Again, as in the case of single infections, *T. buffeli *was also the most prevalent species in mixed infections. It appeared in 11 of them (78.6%), in combinations of two or even three, with all the other species except *B. bovis*. This was the only species never found in combination with other piroplasms in our study, although Almeria *et al. *[3] found it in a mixed infection with *Theileria *sp. Opposite to *B. bovis*, *B. bigemina *was only detected as a mixed infection with either *T. buffeli *or *T. annulata *or both. This same situation was also found by Almeria *et al. *[3]. Although the number of samples with mixed infections is relatively small, the association of certain piroplasm species and not others in mixed infections might be linked to the tick vectors present in the vegetation of a certain area.

The 72 samples with positive RLB hybridisation but negative microscopy, corresponded in most of the cases to infections with *Theileria *species (75.0% of *T. buffeli *and 15.3% of *T. annulata*) and to lesser extent with *Babesia *species (13.9%). These samples (except for *B. major *and *B. bovis*) had haematology values within the normal ranges suggesting that they would correspond to animals that have overcome the parasitaemia but remain as carriers of the haemoparasite. These chronic asymptomatic carriers may serve as reservoirs of the infection and therefore be of serious concern in terms of piroplasmosis control. The lower prevalence of *Babesia *species detected among carrier cattle as compared to carriers of *Theileria *spp., also observed in sheep and horses [18,19], would be explained by the fluctuations in parasitaemia that occur during the chronic phase of infection by *Babesia *species [9,28,29] or the low numbers of intraerythrocytic piroplasms in the circulating bloodstream of *Babesia *carriers [30].

## Conclusion

This study revealed that subclinical infections are common and that bovine piroplasmosis does not have a clear pattern that allows clinicians to undoubtedly identify animals with babesiosis or theileriosis, particularly in the latter case, in which clinical signs can be highly unspecific. Therefore, sensitive and specific laboratorial tests like RLB, which clearly identify the parasite and can detect asymptomatic carriers, are essential to establish good control measures and to avoid unnecessary treatments. In the light of the advantages of RLB, a universal macroarray could be designed to identify all possible piroplasms infecting livestock. The present work is the continuation of our previous studies dealing with the genetic diversity, epidemiology and phylogenetic analysis of piroplasms infecting horses and sheep [18,19]. The application of macroarrays to piroplasm discrimination would help to investigate the host susceptibility and parasite-host specificity relationships.

## Methods

### Clinical samples

A total of 263 bovine blood samples submitted between the years 2000 and 2004 to the Diagnostic Service of NEIKER were included in this study: 88 with clinical suspicion of piroplasmosis (hyperthermia, anaemia, and/or haemoglobinuria, icterus, associated to tick infection) and 175 from animals with unspecific signs (anorexia, body weight loss, reduced milk production, abortion and reproductive failure) for which haemoparasites analysis was requested. Samples were collected from 77 farms from the northern half of Spain and two from Southern Spain. The number of samples submitted from each farm varied from 1 to 30 with the following distribution: 1 sample from 38 farms, 2–10 samples from 36 farms and 11–30 samples in 5 farms. Blood samples submitted in EDTA containing tubes were analysed by haematological and parasitological examination, and stored at -20°C until subsequent DNA purification and hybridisation analysis.

### Haematological and microscopy examination

Leukocyte and erythrocyte counting was carried out with an electronic counter (Iber-cell, Barcelona, Spain). Packed cell volume (PCV) was measured by the standard microhaematocrit method, and haemoglobin concentration was determined by a colorimetric assay in a spectrophotometer. Leukocyte differential counting was carried out by thin blood smears stained with Giemsa and analysed under an oil-immersion objective, differentiating at least 100 cells. Giemsa-stained slides were searched for intracellular forms with morphology compatible with *Theileria *or *Babesia *under an oil-immersion objective (100 ×).

### DNA extraction

DNA was extracted using the QIAamp DNA Mini kit (Qiagen, Hilden, Germany) for DNA purification from whole blood and DNA yields were determined with a NanoDrop^® ^ND-1000 Spectrophotometer (NanoDrop Technologies, DE, USA).

### PCR amplification and RLB hybridisation

Prior to reverse line blot (RLB) hybridisation the hypervariable V4 region of the 18S rRNA gene of the genera *Theileria *and *Babesia *was amplified using primers RLB-F (5'-GACACAGGGAGGTAGTGACAAG-3') and RLB-R (biotin-5'-CTAAGAATTTCACCTCTGACAGT-3') (MWG Biotech AG, Germany), as adapted by Georges *et al*. [17]. Reactions were performed in 25 μl volumes as previously described [18,19]. Oligonucleotide probes containing a *N*-(trifluoroacetamidohexyl-cyanoethyl, *N*,*N*-diisopropyl phosphoramidite [TFA])-C_6 _amino linker were synthesised by MWG Biotech AG (Germany). Sequences of the oligonucleotide probes used are summarised in Table 2. The oligonucleotides were diluted in 160 μl of 500 mM NaHCO_3 _(pH 8.4) to a final concentration of 8 μM. Preparation of RLB membrane and hybridisation were carried out as previously described [18,19]. After developing, the PCR products were stripped from the membrane [18,19] and membranes reprobed a maximum of 8 times.

Plasmids including the amplicon of the V4 region of the 18S rRNA gene of each of the six species analysed were constructed as previously described [18,19] and used as positive controls. To monitor for false-positive results, negative controls included during DNA extraction and PCR amplification were subjected to RLB hybridisation.

### Statistical analysis

Haematimetric variables, microscopic and RLB data were submitted to analysis of variance and comparison of means using the GLM procedure on the SAS statistical package (Version 8.0). Comparison of frequencies for testing associations between piroplasm types and clinical disease were carried out with Chi square or Fisher exact tests. *P *values less than 0.05 were considered significant, and quantitative results are presented as mean ± standard error.

## Competing interests

The author(s) declare that they have no competing interests.

## Authors' contributions

JG carried out the experimental work and participated in drafting of the manuscript. DN carried out part of the experimental work and critically revised the manuscript. ALG interpreted data and participated in writing the manuscript. RAJ performed the statistical analysis and participated in the critical reading of the publication. AH participated in the design and coordination of the study and drafted the manuscript. All authors read and approved the final manuscript.

## Figures and Tables

**Table 1:** Distribution and frequency (%) of piroplasm species.

	***RLB***		***Microscopy***	
	
**Piroplasm species**	**N**	**%**	**N pos (anal) **^**a**^	**%**
*T. buffeli*	101	38.4		
*T. annulata*	15	5.7		
Total single infections with *Theileria *spp.	*116*	*44.1*	*50 (111)*	*19.8*
*B. bovis*	6	2.2		
*B. bigemina*	0	0.0		
*B. divergens*	1	0.4		
*B. major*	5	1.9		
TOTAL single infections with *Babesia *spp.	*12*	*4.5*	*3 (11)*	*1.2*
*T. annulata *+ *T. buffeli*	2	0.8		
*T. annulata *+ *T. buffeli *+ *B. bigemina*	2	0.8		
*T. annulata *+ *B. bigemina*	3	1.1		
*T. buffeli *+ *B. bigemina*	2	0.8		
*T. buffeli *+ *B. divergens*	2	0.8		
*T. buffeli *+ *B. major*	3	1.1		
TOTAL mixed infections	*14*	*5.4*	*10 (13)*	*3.9*
NEGATIVE	*121*	*46.0*	*10 (118)*	*3.9*
TOTAL analysed	263		253	

^a^, number of samples positive by microscopy examination (*Theileria *spp. or *Babesia *spp.) of the total number of samples analysed within each group of RLB-identified species.

**Table 2:** Sequence of oligonucleotide probes covalently linked to the membrane.

Probe	Sequence (5'-3')	Tm (°C)	Reference
catchall	TAATGGTTAATAGGA(A/G)C(A/G)GTTG	54.7^a^	[17]
*Theileria *spp.	GTTGAATTTCTGCT(A/G)CAT(C/T)GC	55.9^a^	[19]
*Babesia *spp.	CCT(G/T)GGTAATGGTTAATAGGAA	55.6^a^	[20]
*T. annulata*	CCTCTGGGGTCTGTGCA	57.6	[17]
*T. buffeli*	GGCTTATTTCGG(A/T)TTGATTTT	52.0^a^	[9]
*B. bigemina*	CGTTTTTTCCCTTTTGTTGG	53.2	[9]
*B. bovis*	CAGGTTTCGCCTGTATAATTGAG	58.9	[9]
*B. divergens*	GTTAATATTGACTAATGTCGAG	52.8	[9]
*B. major*	TCCGACTTTGGTTGGTGT	53.7	[17]

^a ^Tm for degenerate oligonucleotides are approximate values
